# CD4: a vital player in the teleost fish immune system

**DOI:** 10.1186/s13567-018-0620-0

**Published:** 2019-01-07

**Authors:** Hassan Ashfaq, Hatem Soliman, Mona Saleh, Mansour El-Matbouli

**Affiliations:** Clinical Division of Fish Medicine, University of Veterinary Medicine, Veterinärplatz 1, 1210 Vienna, Austria

## Abstract

CD4 is a nonpolymorphic transmembrane glycoprotein molecule that is expressed on the surface of T-helper cells and plays an essential role in the immune response. It functions as a coreceptor with the T-cell receptor by binding to major histocompatibility complex class II on the surface of dendritic cells that present antigens. CD4^+^ T cells hold a key position in coordinating the immune system through production of several cytokines after activation and differentiation. The CD4^+^ T helper subtypes (T-helper 1, T-helper 2, T-helper 17, T-helper 9, and regulatory-T cells) perform different immune functions subsequent to their differentiation from the naive T cells. Different types of CD4^+^ T cells require different cytokines such as drivers and effectors, as well as master transcription factors for their activation. Fish cells that express CD4-related genes are activated in the presence of a pathogen and release cytokines against the pathogen. This review highlights the types of CD4^+^ T cells in fish and describes their direct role in cell-mediated and humoral immunity for protection against the intracellular bacterial as well as viral infections in fish.

## Introduction

The fish immune system is categorized into innate and adaptive immunity. Adaptive immunity is the subsequent defense barrier of the immune system that pathogens encounter if they overcome the physical barrier and the other elements of innate immunity [1]. The adaptive immune system identifies pathogens with the help of molecules that are generated via somatic mechanisms through the generation of B- and T-lymphocytes [2]. In fish, cell-mediated immune response is governed by different types of leukocytes including T-lymphocytes, which comprise cytotoxic T-lymphocytes (CTLs) and T helper cells (T_h_) [3]. These cells express different cell markers which allow distinguishing among them [4]. The initiation of a response to an antigen is always presided over by a specific receptor known as the T cell receptor (TCR), which demarcates the T-cells from the other lymphocytes [5]. CD4 is a transmembrane glycoprotein expressed on the surface of T_h_ cells and plays an essential role in the immune response. T-helper cells that express CD4 (CD4^+^ T_h_ cells) coordinate the immune response by acting either as effector cells or as memory cells [6]. CD4^+^ T_h_ cells usually perform the “helper” cell functions [7]. In fish, CD4^+^ T cells’ function is thought to be comparable to that of mammals due to the presence of TCRs, CD4-like genes with the same number of domains (D1-D4) and an important structural factor such as Lck Motif [8]. The characterizations of CD4^+^ cell populations and functions have been defined due to the availability of suitable markers for T-lymphocytes in fish. CD4^+^ T_h_ cells are essential for triggering and maintaining both natural and vaccine-induced immunity [9]. This review discusses the importance of CD4^+^ T helper cells and their role in adaptive immunity, focusing on their types, characterization, expression, differentiation pattern, operational mechanism, and signaling to the other components of the immune system in fish.

## Functions of CD4^+^ T cells in fish

**Figure 1:**
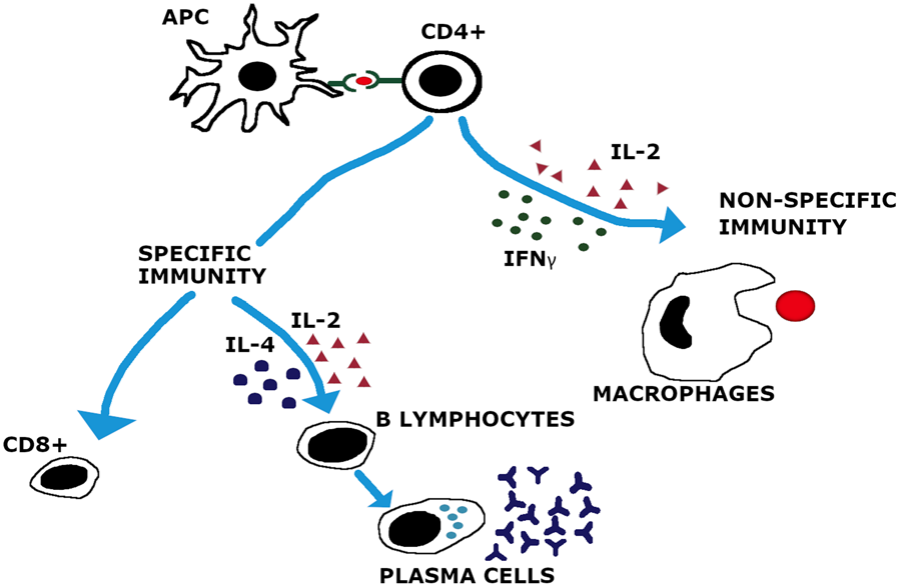
**CD4 cells orchestrating immune response.** Recruitment of CD8, B lymphocytes and Macrophages by CD4 cells and production of different interleukins and interferon’s to carry out desired protection against various organisms, equally important for specific and non-specific immunity. Assembly of inflammatory cytokines in fish with infection is triggered, subject to leukocyte mobilization. Activated T-helper cells with CD4 co-receptor, produces IL-2 and IFN-γ to encourage macrophage activation for a general non-specific immune response. Likewise, IL-2 and IL-4 elicit response of B lymphocytes for specific immunity.

Initiation of T-cell response requires complex cellular interaction involving both polymorphic and non-polymorphic regions of TCR, mediated by MHC class I and II molecules and enhanced by their coalition with T cell co-receptors i.e., CD4 and CD8 [10]. Surprisingly, the MHC class II and CD4 related genes were observed to be missing in only one fish type, the Atlantic cod, possibly because of a genetic modification. As far as the functional organization of the fish thymus is concerned, it is perhaps analogous to the thymus in mammals; this is inferred considering the expression sites of the recombination activating gene (RAG) [11]. CD4^+^ T cells, particularly in fish, have been investigated in several studies. These CD4^+^ T cells accomplish several functions in fish (Figure 1), such as stimulating macrophages to boost microbicidal activity and B-cells to produce antibodies, as well as enhancing cell-mediated immunity [12]. These cells also support the employment of neutrophils, eosinophils, and basophils to the inflammation site, the antigen-specific proliferation, maneuvering the immune response, and the regulation/suppression of the immune responses [13], thereby establishing a base for a superior overall immune response. Adaptive immune response relies on the stimulation by T-helper cells, which express several cell-surface markers, among which CD4 is the most effective marker to delineate the T-helper subsets [14]. CD4 is not expressed by the other adaptive immune cells such as CLT, however, it is also expressed by a few subsets of dendritic cells and macrophages. CD4 was reported to be connected through an interface with an antigen (e.g., bacteria) of extracellular origin in ginbuna carp [15]. In viral infection, a skewed immune response triggered by CD4^+^ cells via upregulation of IL-12 cytokine through stimulation by CTL was observed in the common carp [4]. Similarly, in extracellular parasitic infection, higher expression of cytokine genes related to a particular CD4^+^ cell was observed in fugu [13].

## Molecular characterization of CD4 in fish

Mammalian CD4 belongs to the immunoglobulin superfamily and contains four Ig-like domains (D1-D4) and important cytoplasmic tail linked with p56lck or tyrosine kinase protein [16]. Two of the CD4 Ig-like domains are constant type-C (D2 and D4) and the other two are variable type-V (D1 and D3) [16]. Generally, teleost fish contain two CD4 genes; CD4-1 contains four Ig-like domains, and CD4-2, contains two or three Ig-like domains. The p56lck motif in their cytoplasmic domain is responsible for intracellular signaling, resulting in a series of phosphorylation cascades, defining helper activity of the T cell, after interaction with an antigen [17]. These basic components (domains and cytoplasmic motifs) are important for primary processing and activation of T helper cells in rainbow trout after interaction with antigen as in mammals [8]. It has been established that immunoreceptor tyrosine-based activation motif (ITAM) acts as a bridge between TCR and internal cellular machinery [17].

Most fish species contain two types of CD4-like molecules, CD4-1 and CD4L-2 (or CD4REL) [8]. In rainbow trout, although the four Ig-like domains are comparable to those in the mammalian CD4 glycoprotein, the first domain lacks the cysteine (Cys) residue encoded by 12 exons [8] instead of 10 in humans [16] and birds [18]. The non-polymorphic region of MHC class II interacts with human CD4 mainly through strands of the D1 domain via amino acids [19]. Likewise, a similar D1 domain structure and only one Cys residue, instead of two in mammals and birds, were reported in fugu and many other fish species [20]. Additionally, the MHC II region binding to trout CD4 were found to be conserved between fish and mammals [21]. The role of the Lck motif has been very well studied in mammals, and its presence in fish may be suggestive of a similar role [20]. The dileucine motif in the cytoplasm, which is also important for the activation of T-cells was, however, found to be absent in both trout and fugu CD4 sequences, suggesting a slight variation in activity [8]. Table 1 gives a summary of the information regarding the characterization of CD4 in particular fish species (Table 1).

**Table 1:** **CD4 features in fish.**

Species	Number of CD4+ homologs	Types	(IG)-like domains	ORF	Gene accession number	References
Rainbow trout	2	tCD4 CD4REL CD4L2a 1,2 CD4L-2b	Four Two	489 334 279.293 315	AY973030 AY973031 AY899931,33 AY899932	[8] [23]
Atlantic salmon	3	CD4-1 CD4-2a, CD4-2b	Four Two	490 311.315	EU585750 EU585752 EU585751	[20]
Catfish	2	IpCD4L-1 IpCD4L-2	Four Three	471 412	DQ435301 DQ435302	[24]
Common carp	1	CD4L	Four	458	DQ400124	[27]
Sea bass	1	CD4	Four	480	AM849811	[25]
Fugu	2	CD4L-1 CD4L-2	Four Two	463	AB16405 AB164055	[22]
Ginbuna carp	2	CD4L-1a CD4L-1b	Four Four	464 263	AB331216 AB331217	[26]
Atlantic halibut	2	CD4-1 CD4-2	Four Two	462 308	FJ185042 GU985449	[28] [29]
Japanese Flounder	2	jfCD4-1 jfCD4-2	Four Two	464 302	AB643634 AB716324	[3]

Two CD4 homologs have been documented in rainbow trout (*Oncorhynchus mykiss*), one (tCD4) with the standard four Ig-like domains, and the other (tCD4REL) with only two domains [8]. The rainbow trout CD4, tCD4, has a CXC chemokine receptor motif in its cytoplasmic domain which is involved in lymphocyte-specific protein tyrosine kinase (Lck) binding. A function similar to that in mammals has been suggested [8]. It is also reported that tCD4REL has the CXC motif serving as a binding site for p56LCK, suggesting the same function as CD4-1 in other fish species. The tCD4 encodes an open reading frame (ORF) of 489 amino acids, while tCD4REL encodes an ORF of 325 amino acids, sharing 90% nucleotide identity with each other. The sequence alignment of tCD4REL with CD4 sequences from other species showed great similarity with regards to D1 and D2 domains, suggesting the same phenomenon of interaction with the MHC class II protein. Both tCD4 and tCD4REL have sites for glycosylation (N and O types), similar to other species. The N-terminus of fish Lck encompasses a CXXC motif and numerous other hydrophobic residues at the same position as in the mammalian Lck, indicating the interaction between CD4L-2 and Lck in fish. Like any other fish species, conserved Cys in the F strand of the D1 domain was identified but the Cys residue in the B strand of D1 (conserved in mammals) was absent for the disulphide bond in the rainbow trout CD4 sequence [8].

Fugu (*Takifugu rubripes*) CD4 gene encodes an ORF of 463 amino acids and contains four Ig-like domains (D1–D4), a cytoplasmic domain, and a transmembrane region [22]. It contains four N-linked glycosylation sites in the D4 domain. In mammals, the disulfide bond in D1 and D2 is essential for binding with the MHC class II molecule; this bond is absent in the first two domains of fugu. However, a distinct type of disulfide bond at D3 is suggestive of a mechanism of interaction different than that in mammals. However, the tyrosine kinase motif p56lck (lck motif) present in the cytoplasmic domain, which is involved in T-cell maturation and signaling, is perceived to be conserved. A second CD4-like gene (CD4L-2), orthologue to the trout (CD4L-2) gene, with two Ig-like domains both (VC) type and similar cytoplasmic tail (Y-C-Q-C) motif for Lck binding was identified [23].

In Atlantic salmon (*Salmo Salar*) three CD4 genes were reported; CD4-1, CD4-2a, and CD4-2b [20]. A comparison of these genes with the documented teleost CD4 molecules affirmed the conserved cysteine sequences along with four extracellular Ig-like domains in CD4-1, two extracellular Ig-like domains in both CD4-2a and CD4-2b, and a cytoplasmic domain with Lck motif-binding sites in all three homologs. The sequences in CD4-2 do not contain motifs for glycosylation, particularly of the N-linked nature; nevertheless they contain numerous motifs for O-linked glycosylation indicating a different structure with the same function as in mammals.

Two CD4-like molecules, CD4L-1 and CD4L-2, are present in channel catfish (*Ictalurus punctatus*), sharing 19% amino acid identity with each other [24]. Similarly to mammals, the CD4L-1 gene encodes a protein containing four extracellular Ig-like domains and exhibits similar gene organization, while the CD4L-2 gene encodes a protein containing just three Ig-like domains and exhibits a genetic organization different than that in mammals. Both CD4L-1 and CD4L-2 contain a cytoplasmic motif with p56Lck-binding sites and four N-linked glycosylation sites. It was reported that the catfish CD4 gene sequence is diverse to the other fish CD4 sequences since it contains only three Ig-like domains and CD4L-2 has only 20–24% amino acid similarity with the CD4L of other fish species. Exon–intron arrangement in CD4L-1 appears to be similar to that of the mammalian and avian counterparts, while the CD4L-2 gene arrangement is completely different.

In sea bass (*Dicentrarchus labrax*), four Ig-like domains (D1-D4) are present, however, the number of Cys residues is different among all the domains, i.e., D1 contains one Cys residue (Cys114), while two Cys residues are present in D2 (Cys193 and Cys156), D3 (Cys233 and Cys316), and D4 (Cys355 and Cys404) [25]. Sea bass CD4 encodes 480 amino acids and contains the typical four Ig-like domains with cytoplasmic CXC Motif similar to mammals. It exhibits 40%, 30% and 33% of amino acid identity in rainbow trout, carp, and catfish, respectively and 23% with humans.

The CD4 homologs of ginbuna crucian carp (*Carassius auratus langsdorfi*) are of two types, namely CD4L-1a and CD4L-1b, sharing 95% identity with each other in terms of amino acid profile [26]. It was reported that both CD4L-1a and CD4L-1b contained four Ig-like domains (D1-D4), a cytoplasmic domain, and a transmembrane domain, similar to the CD4 from the other species. Phylogenetic investigation specified greater closeness of ginbuna crucian carp CD4 to the teleost CD4L-1, compared to teleost CD4L-2.

A single CD4 homolog, CD4L, was identified in common carp (*Cyprinus carpio* L) [26]. It contains four extracellular Ig-like domains, a transmembrane region, a cytoplasmic tail, a conserved tyrosine kinase motif p56lck and 21 phosphorylation sites. CD4L does not have a Cys residue for a disulphide bond, similar to fugu CD4, rainbow trout CD4L-1, channel catfish IpCD4L-1, and the D1domain in carp [26].

The Atlantic halibut (*Hippoglossus hippoglossus*) CD4-1 was reported to contain four Ig-like domains (D1-D4) [two V-types and two C-type] that were extracellular in nature and structurally analogous to the other documented CD4 in fish and mammals [28]. It contain three O-linked glycosylation sites at D3, three N-linked glycosylation sites similar to the conserved ones in almost all fish species excluding the zebrafish, and a CXC motif comparable to that in the other teleosts and mammals. A second halibut CD4-2 molecule was reported, which contained two Ig-like domains with three O-type glycosylation sites [29].

In Japanese flounder (*Paralichthys olivaceus*), two CD4 homolog were identified; jfCD4-1 and jfCD4-2 [3]. The first gene (jfCD4-1) encodes an ORF of 464 amino acids. It contains classical four Ig-like domains and an Lck binding motif in the cytoplasmic domain. Cysteine is absent in the first domain of the jfCD4-1 as in the other known CD4 from teleosts. It contains four N-linked glycosylation sites. However, jfCD4-2 encodes an ORF of 302 amino acids and contains two Ig-like domains with a conserved CXC Lck binding motif.

## Antibodies used to study the expression of CD4^+^ T cells in fish

Monoclonal antibodies (mAbs) against trout CD4-1 and CD4-2 were generated and validated to study the surface expression of CD4-1 and CD4-2 molecules in trout leukocytes [30]. Similarly, mAbs against teleost CD4 were produced in clonal ginbuna crucian carp (*Carassius auratus langsdorfii*) to investigate the functions of CD4 positive T cells [31]. Accordingly, it was suggested that the presence of CD4-positive T cells in the ginbuna crucian carp is the equivalent of the helper T-lymphocyte in mammals. Additionally, mAbs directed against thymocytes of rohu, (*Labeo rohita*) was developed and characterized [32]. This mAbs emerged to be a suitable marker for T-lymphocytes and could be a valuable tool in studying the immune response and ontogeny of the rohu immune system.

Furthermore, a polyclonal antibody against zebrafish (*Danio rerio*) CD4 (zfCD4-1) was developed and used to investigate whether CD4-1^+^ lymphocytes can express typical T_h_ cytokines following antigen specific stimulation [33]. The results of this endeavour revealed that zfCD4-1^+^ lymphocytes induce the expression of cytokines and master transcription factors relevant to T_h1_/T_h2_-type responses as a consequence of boosting with specific antigen [33]. Specific anti-fugu CD4 antibodies (Abs) were produced to isolate CD4^+^ T cells from Japanese puffer fish, *Fugu rubripes*, and characterize their cytokine expression profile [34]. It was found that several distinct T_h_ cytokines were expressed in fugu CD4^+^ T cells and these cells expressed T-cell marker genes but not macrophage or B-cell marker genes. Based on the results of these experiments, it was suggested that T_h_ subsets exist in fish and that the orientation of immune responses is regulated by T_h_ cytokines expressed from the cells, as occurs in mammals.

## CD4^+^ T cell subtypes

**Figure 2:**
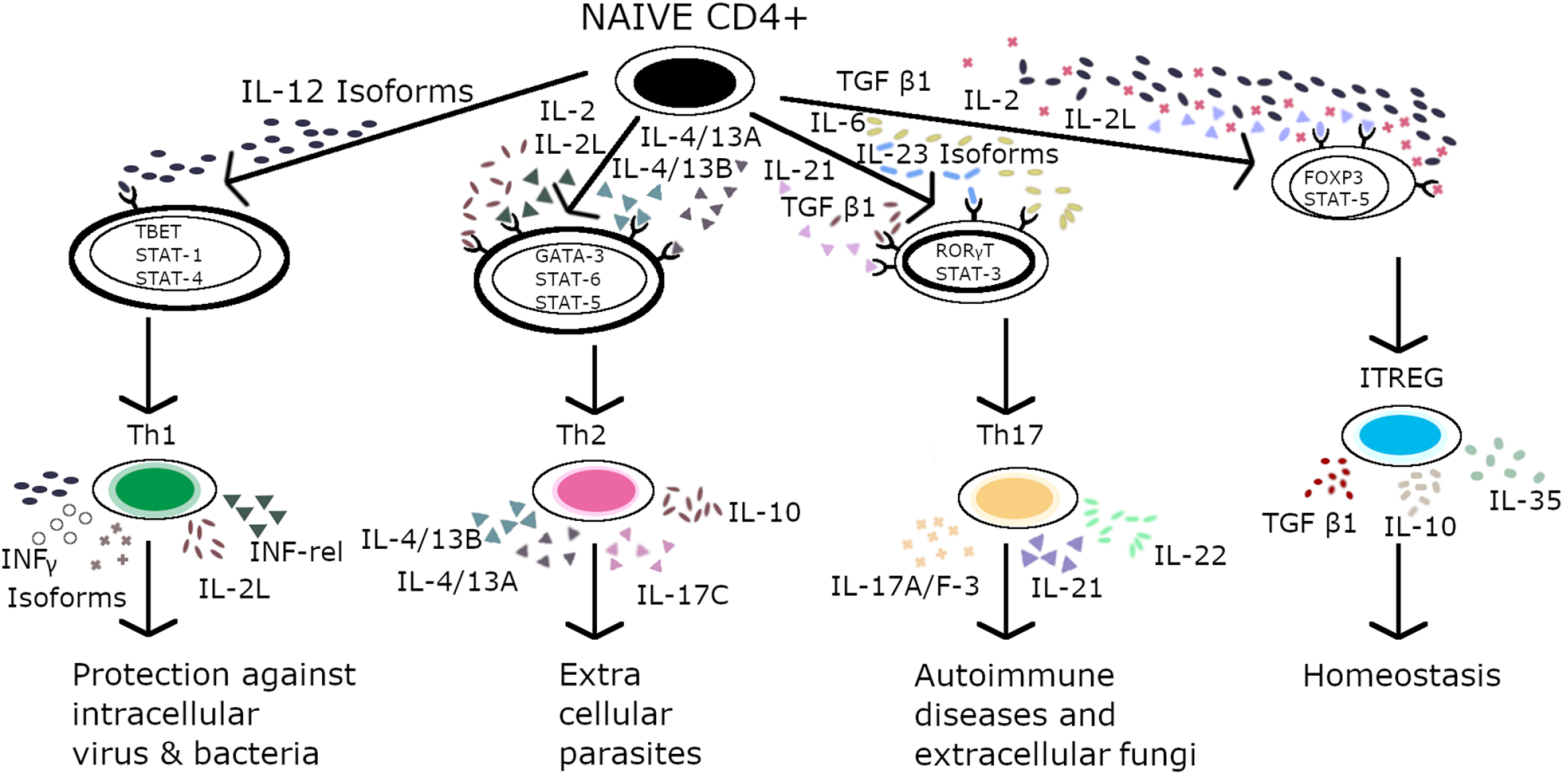
**Summary of expected CD4+ cell differentiation in fish.** Expected Milieu of cytokines (denoted by colored symbols) influencing CD4+ T cell differentiation with (IL-12) causing naïve CD4 cells ultimately differentiated into Th-1 cells to get rid of invading intracellular pathogen i.e. Bacteria, Viruses. Similarly, IL-2, IL-2L, IL-13A, IL-13B brings forth differentiation of Th2 cells in response to extracellular parasitic infectious agent. Differentiated Th-17 cells; autoimmunity and I-Treg cells; homeostasis are produced in response to cytokines IL-23, IL-21, IL-6 and IL-2, IL-2L respectively with TGF-ß1as a common precursor.

Subsequent to interaction with the antigen-MHC complex, the naive CD4^+^ T cells get activated and differentiate into specific subtypes, namely, T-helper 1, T-helper 2, T-helper 9, T-helper 17, and induced regulatory-T cells, each with a characteristic cytokine profile [35]. The mechanism through which the CD4^+^ cells tackle different pathogens has been elucidated previously. T_h_1, T_h_2, T_h_17, T_h_9, and iTreg cells delineate the division of labor of the CD4^+^ cells (Figure 2) for governing a particular immune response [9]. This control over the immune system is based on the production of different types of cytokines, such as IFN-α, TGF-β-1, IL-4, and IL-17, similar to mammals [6]. Each CD4^+^ T-helper cell possesses a characteristic ability to respond to a particular cytokine (inductive) and processes it via selective master transcription factors, and in turn produces another set of cytokines (functional) in order to perform its roles [36, 37]. Studies investigating cytokines, particularly the interleukin (IL) category, in fish have been reported and published previously [38]. When a pathogen intrudes into the body, the T helper cells-related cytokines are generated, which then modulate the inflammatory signals to control phagocytes and annihilate the invading antigen. These cytokines also regulate the antigen-presenting cells (APC) in order to commence the adaptive immune response [39, 40]. The differentiation of different lineages of immune cells depends on the complex network of specific cytokine signaling and transcription factors, followed by epigenetic modifications [41, 42].

## Potential factors for differentiation of CD4^+^ T cell subtypes in fish

### T_h_1 cell differentiation

The T-cell differentiation process is primarily initiated by cytokine signals generated by APC subsequent to the encounter with pathogens [43]. In mammals, IL-12 and interferon (IFN-γ) are the key cytokines responsible for T_h_1 differentiation, through the regulation of several transcription factors including the T-bet master regulator, signal transducer and activator of transcription-1 (STAT-1), and STAT-4. Subsequent to antigen interaction, APC (mostly dendrites) release huge amounts of IL-12, which is captured by the naive CD4^+^ T cells, following which IFN-γ is released [14]. Within the site of both IFN-γ and IL-12, STAT-1 and STAT-4 trigger T-bet expression. T-bet subsequently invigorates the IFN-γ production by the differentiated T cells, consequently amplifying the T-bet expression and strengthening the selective differentiation of T_h_1 cells via the expression of IL-12 [44]. T-bet, also known as Tbx 21, is responsible for type-1 (cell-mediated) immunity in almost all the immune cells of the innate as well as specific immunity. The T-bet gene has been identified in rainbow trout [45], grass carp [36], and zebrafish [37]. In an investigation involving rainbow trout, it was established that T-bet is expressed equally in the spleen and head kidney; although a strong expression of T-bet has been reported in the peripheral blood leukocytes, spleen, and head kidney following bacterial and parasitic infections. It was suggested that T-bet plays a crucial role in performing in cell-mediated immunity in the teleost fish, as its expression was found to be upregulated with the use of T cell-stimulant phytohemagglutinin (PHA). In fish, multiple isoforms of IL-12 [not just single IL-12] and IFN-γ-rel [instead of IFN-γ] are considered to be the main cytokine drivers for a possible T_h_1 differentiation; also, two particular isoforms, viz., IL-2 and IL-2L, have been documented to date [13, 46]. However, the master transcription factors are considered to be the same in fish and mammals both, for example, T-bet along with its assisting transcription factors STAT-1 and STAT-4 [47].

IL-12 (heterodimeric cytokine with p35 and p40 subunits) is a type of interleukin produced mostly by macrophages, dendrites, and neutrophils subsequent to interaction with an antigen; this cytokine affects the CD4^+^ T_h_1 cell differentiation. IL-12 possesses the ability to increase the cytolytic activity of natural killer cells and T cells [48]. Fish p35 and p40 subunits were first identified in fugu [13]. In common carp and sea bass, the single p35 gene has been documented. The p35 subunit of IL-12 is not as expressive in fish as it is in mammals, with restricted expression at sites such as the thymus and gills and unrestricted expression in blood, head kidney, and spleen. However, following viral and bacterial exposure, the expression of the p35 subunit was reported to be upregulated in the common carp [27]. It was also propounded that the p40 gene in fish exhibits different isoforms, viz., 40a, 40b, and 40c, and that the p40a isoform demonstrates greater homology to the mammalian gene compared to the other isoforms. It was observed that the p40 subunit of IL-12 was expressed more in all tissues compared to the p35 subunit; the expression of the isoforms of p40 differed in different tissues following a viral infection, and p40c was not expressed in the thymus of fish [4]. The presence of IL-12 isoforms may propound the necessity of additional studies in order to investigate these different isoforms and explore the ways in which these isoforms successfully control T_h_1 differentiation as well as the other functions involved in the fish immune response.

IFN subtypes produced by the CD4^+^ T_h_1 cells are imperative for both adaptive and acquired immunity, as they provide the antiviral ability to the cells. There are three classes of IFN-I, II, and III; fish IFN-γ belongs to class II. The IFN-γ gene was first reported in fugu, [49], followed by zebrafish [50] and Atlantic salmon [51]. Teleost-specific IFN-γ-rel reported in zebrafish and fugu was observed to be different in comparison to IFN-γ in the other fish species [13]; consequently, two subtypes of IFN (IFN-γ and IFN-γ-rel) have been documented in bony fish.

Although the exact evidence of the presence of T_h_1 differentiation in fish has not been completely established, the upregulation of IFN-γ, IL-12, and T-Bet expression subsequent to the induction of the infection model or vaccination in rainbow trout, and the correlation of T-bet expression with IFN-γ production indicates events associated with T_h_1 regulation.

### CD4^+^ T_h_2 cell differentiation

Unlike the T_h_1 cells that are responsible for type-1 immunity, T_h_2 cells stimulate the production of antibodies by inducing IL-4, IL-10, IL-13, and IL-25, which are accountable for the proliferation of B lymphocytes [52]. It has also been observed that in the presence of parasitic infestation or venoms, IL-5, IL-9, and IL-13 are released, stimulating the mast cells, and a cascade of allergic reactions that follow. The strength of the signals via TCR is essential as it governs the differentiation between T_h_1 and T_h_2 cells; stronger signals initiate T_h_1 differentiation, while weaker signals cause T_h_2 differentiation [53]. According to investigations, low-frequency signals cause the T-cells to promptly induce the expression of T_h_2 master regulatory transcription factor GATA3, which leads to IL-2 production. This eventually activates STAT-5 via IL-4Ra expression and IL-4 is produced as a result of the dimerization of STAT-6 caused by STAT5 and GATA3 activation. STAT-5 activation may drive the effector cytokines to be fully expressed, resulting in T_h_2 cells; therefore, IL-4 is the key cytokine for T_h_2 cell development. Similarly, in fish, two IL-4-like genes have been documented in tetraodon [38]. Since these genes were related to IL-13, they were named IL-4/13A and IL-4/13B [54]. These isoforms are considered the precursors for T_h_2 cell development and antibody production, just as in mammals. IL-4/13A induces the production of immunoglobulin-producing cells in zebrafish, causing antibody production. Similarly, two isoforms of IL-2 have been isolated from fish, namely, IL-2 and IL-2L. IL-2 has been discovered in lower vertebrates and the existence of the IL-2 gene was recognized through genome analysis [55]. The authors reported that in response to mitogen PHA, trout IL-2 was drastically upregulated in the head kidney leucocytes in a mixed-lymphocyte reaction controlled by STAT-5 transcription factor. In mammals, an IL-7-like cytokine, known as the thymic stromal lymphopoietin (TSLP), has been identified. It causes IL-4 to induce the development of T_h_2 cells. So far, there has been no evidence of the TSLP gene in fish, inviting fresh research initiatives to explore novel genes involved in T_h_2 differentiation. Similarly, IL-25 and IL-17 are also considered to have a role in the activation and differentiation of T_h_2 cells, through the production of IL-4 and by exhibiting an inhibitory effect on the T_h_1 cells [40]. Mammalian IL-25/IL-33 has not yet been detected in any of the teleost fish species. In addition to STAT-3, STAT-5, and STAT-6, the transcription factor GATA3 is a master regulator for T_h_2 differentiation, and its expression is decreased during T_h_1 differentiation. STAT-5 requires a signal from IL-2 via IL-4Ra expression, and STAT-6 requires IL-4 for its activation. In CD4^+^ T cells, GATA3 is responsible for the downregulation of IFN-γ and T_h_1 differentiation, in addition to its regular functions of T_h_2 development and cytokine production [56]. GATA3 has been identified and isolated from different species of fish, including zebrafish [57], salmonids [58], and grass carp [36]. Interestingly, the expression of GATA3 and T-bet in trout was increased by PHA, signifying their presence in the activated T cells, particularly in the spleen, following the induction of infection [59]. Certain bacteria (e.g., *Yersinia ruckeri*) were able to downregulate the production of both GATA3 and T-bet in fish through mechanisms that suppressed the host immune system [45]. In summary, the presence of IL-4/13 and GATA3 along with STAT5 and STAT6 in fish emphasizes the requirement for further investigation on T_h_2 differentiation.

### CD4^+^ T_h_17 differentiation

T-helper 17 cells (T_h_17 cells) have been reported to be important for the prevention of autoimmune disorders, due to their ability to serve either as protective/non-pathogenic cells or proinflammatory pathogenic cells. T_h_17 cells are characterized by IL-17 production. T_h_17 cells are considered to be closely related to iTreg cells, and the differentiation of these two cells is inversely proportional. T_h_17 cells are the protective cells that maintain and guard the mucosal surface against microbial populations. IL-6, IL-21, IL-23, and TGF-β are responsible for the segregation of T_h_17 cells, along with the key precursors such as retinoic acid receptor-related orphan receptors gamma (RORγ) and alpha (RORα) and the signal transducer and activator of transcription 3 (STAT3) [41]. IL-6 and TGF-β stimulate the production of a non-pathogenic type of T_h_17 cells, while IL-23 and IL-1β are accountable for the production of a pathogenic type of T_h_17 cells [39]. The main effectors or signature cytokines IL-17A, IL-17F, IL-21, and IL-22 are responsible for all the functions of the T_h_17 cells [40]. IL-21 secreted by T_h_17 cells during differentiation collaborates with TGF-β to further increase the production of STAT3-dependent IL-17 and the expression of IL-23R. IL-23 is necessary for the expansion and continuance of the T_h_17 population.

T_h_17 cells produce IL-17A and IL-17F, and their expression is controlled by RORγt and STAT3 [41]. RORγt is stimulated by either IL-6 or IL-21, with the help of TGF-β, and for the activation of STAT3 also, IL-23 is required along with IL-6 or IL-21 [42]. An orthologous gene RORγ, instead of RORγt, has been reported in rainbow trout [60]. Three isoforms of IL-17A/F genes, namely, IL-17A/F1, IL-17A/F2, and IL-17A/F3, have been identified in zebrafish [61], rainbow trout [62], fugu [63] and turbot (*Scophthalmus maximus*) [64]. The basal expression of these three isoforms has been reported to be different on the tissue level. IL-17A/F1 in turbot was reported to be highly expressed in head kidney, intestine, and gills. While in trout, IL-17A/F2 was observed to be expressed less in the head kidney in comparison to the gills and intestine [62].

Similarly, the IL-6 gene has been reported in the rainbow trout [65], Japanese flounder [66] and other teleosts [67]. In the above-mentioned publications, the IL-6 gene expression was upregulated following the immune stimulation, compared to normal conditions, in the spleen and brain of rainbow trout. IL-6 in trout was able to promote macrophage growth through the induction of phosphorylation of STAT3 formed during the events of the inflammatory response.

IL-21 has been reported to be expressed by activated T cell and is not present normally in the tissues; it has been indicated to cause T_h_17 differentiation via IL-23R expression [68]. IL-21 has been discovered in fugu [69], rainbow trout and other teleosts [70]. Unlike the mammalian IL-23, multiple isoforms of IL-23 have been reported in zebrafish [69].

Most of the elements of mammalian T_h_17 cell differentiation machinery are also present in fish, including the master transcription factors such as IL-6, TGF-β-1, IL-21, and IL-23, which is suggestive of the presence of T_h_17 cells-like response in fish as well.

### CD4^+^ regulatory T cell differentiation

As the name suggests, regulatory T cells (Tregs), also known as suppressor T cells, are the subset of helper T cells that regulate the immune response, preserve the tolerance of internal structures to self-antigens, and provide protection against autoimmune diseases [71]. It has been perceived that the cytokines TGF-β and IL-10 are responsible for the differentiation of Treg cells as well as for Treg homeostasis [72]. Forkhead box P3 (FoxP3) expression factor is a crucial asset that determines the natural functions of Treg cells [71]. Tregs were first documented along with FoxP3 expression factor in 2003 and were perceived as essential for the inflammatory responses [73]. In brief, Treg cells control the enormity of immune responses to infectious agents and tumors. Similar to the mammalian TGF-β family, different TGF-β isoforms (e.g., TGF-β-3) exist in fish. Fish also contain TGF-β-1, which was first reported in rainbow trout [69]. Besides IL-6, TGF-β-1 also causes T_h_17 cell differentiation, in addition to maintaining the inflammatory environment, suppressing the differentiation of T_h_1 and T_h_2 cells, and initiating the FoxP3^+^ Tregs to defend against autoimmune diseases [74]. Alongside the documentation of all the three isoforms of TGF-β (e.g., TGF-β-3) in fish [75], another gene type TGF-β-6 has been identified in gilthead sea bream. Although IL-10 is produced by all types of CD4^+^ cells, Treg cells are the key source of this cytokine [72]. Interleukin-10-type gene was first recognized in fugu [76], and to date, its two isoforms IL-10a and IL-10b have been identified in rainbow trout [77], zebrafish [78], sea bass [79], grass carp [80], and goldfish [81]. The master transcription factor of Tregs (FoxP3) is required for the immunosuppressive activity and fitness of Tregs. The inducible FoxP3 has been discovered in salmonids [59], grass carp [80], and tetraodon [82], with a little dissimilarity with the mammalian FoxP3 gene which is suggestive of a different mechanism of regulation in fish.

## CD4^+^ T cell response against pathogens

On the basis of the documented cytokines in fish, the expected cytokine production may be hypothesized using the already-available mammalian paradigm. As stated earlier, cytokines such as IFN-γ, IL-4/13, IL-2, IL-17A/F, and IL-10 in fish share homology with the mammalian IL-13, IL-4, IL-17A, and IL-17F, respectively. In fish, a wide range of disorders prevails, several of them infectious in nature, and others being autoimmune disorders [83]. When the antimicrobial activity of the T_h_1 and T_h_2 cells was first documented, these cells were perceived to manage infection, limiting its growth and disastrous effect [84]. T_h_1 cells govern the protective response against the intracellular pathogens such as bacteria, fungi, and protozoa; while, with viruses, the immune response is governed by B-lymphocytes and CD8^+^ cells [84]. T_h_1 cells are effective against the intracellular antigen, causing the release of IFNγ and IL-2 [14]. Therefore, the same may be expected in the fish immune system, considering the aforementioned presence of T_h_1 cytokines, as cytokines are the markers for T_h_1 characterization. In mammals, IL-12 triggers the immune response against intracellular pathogens, causing T_h_1 polarization and alerting the other components of the immune system [84] to respond accordingly; the same may be expected in fish, however, with the IL-12 isoform inducing the IFN-γ isoforms and executing the signaling. This response to a pathogen may cause a little pathological inflammation, which is controlled by iTreg cells that produce TGF-β-1 and IL-10 in order to downregulate the T_h_1 activity. This phenomenon is equivalent to that observed in mammals. In helminths, T_h_2 cells act as officers in command, activated by IL-2 and IL-4 [different isoforms of these cytokines in fish], producing the driver cytokines IL-10 and IL-13 [different isoforms of these cytokines in fish] to activate the eosinophils and mast cells and to induce the production of IgE (T_h_2-dependent antibodies) for the elimination of the invader [84]. T_h_17 cells are involved in protection against the extracellular bacterial or fungal infection. Treg cells regulate the actions of T_h_1, T_h_2, and T_h_17 cells, and are responsible for peripheral tolerance [43]. Treg cells, also known as CD4^+^ CD25^+^ FoxP3^+^ regulatory T cells, are accountable for the insensitive immunological response to self-antigens, expression of foxhead box p3 (foxp3), and the maintenance of the host immune response to a favorable/healthy level. Different phenotypes of Treg cells, namely, CD4-like-2^+^, CD25-like^+^, and Foxp3-like^+^, have been reported in pufferfish [85].

## Conclusions

This review discusses the possible existence of CD4^+^ T_h_ cell differentiation in fish, as well as the molecular characterization of CD4 based on the documented facts. The mammalian paradigm for T-helper subset segregation appeared to have rather a similar machinery and cytokines, although these are just dubious evidence. The adaptive immune system in fish is imperative for both natural immunity and vaccine-induced immunity; therefore, greater knowledge regarding this system will enable achieving greater protection against a particular organism. The efficiency of vaccine development is influenced largely by the production of cytokines, which are considered the most suitable markers for the evaluation of a particular vaccine. Therefore, this review paves the way for the development of strategies for monitoring a strong immune response against a particular pathogen. It is time to utilize our acquaintance with the T cell subpopulation, and develop a novel therapeutic approach for fish, just as in mammals. This review also provides insights into the cytokine network necessary for an early adaptive immune response.

The molecular characterization of CD4 has led to the awareness that different domains and subtypes of CD4 are present in fish, similar to mammals. If the mechanism of antigen binding is compared, mammals contain binding sites that are different from those in fish, although the cytoplasmic Lck and the Ig-like domains are nonetheless present. It may be hypothesized that fish share the same mechanism of antigen attachment as in mammals, with only minor differences. Molecular characterization of CD4 in fish offers imminent advancement of research, through gene expression data, in order to identify the cell-surface markers and proteins coding for T_h_ populations. Further research is required to accurately understand the pathways that lead to the creation of these CD4^+^ T_h_ cells as well as the preferential expansion of the T-cell subset capable of mediating a protective response. Finally, we have to point out that even though there are similarities between fish and upper vertebrate immune regulatory networks, there is not nearly as much hard evidence to support speculative statements and research requests.

## Abbreviations

APCS: antigen-presenting cells
RAG: recombination activating gene
CTLS: cytotoxic T-lymphocytes
TH: T helper cells
GATA: proteins recognize G-A-T-A nucleotide/types
IFNs: interferon types
ROR: retinoic acid receptor-related orphan receptors gamma
T-BET: transcription factor
STAT: signal transducer and activator of transcription-types
TCR: T-cell receptor
MHC: major histocompatibility complex
TSLP: thymic stromal lymphopoietin
CXC: chemokine receptor
FOXP3: forkhead box P3 transcription factor
CYS: cysteine
IG: immunoglobulins
MALT: mucosa-associated lymphoid tissues
IL: interleukins
ITREG: induced regulatory T cells
LCK: lymphocyte-specific protein tyrosine kinase
T-BOX: transcrpition factor
PHA: phytohaemagglutinin
TGF-ß1: transforming growth factor beta 1
